# Echocardiographic Predictors of Mortality in Patients with Pulmonary Hypertension and Cardiopulmonary Comorbidities

**DOI:** 10.1371/journal.pone.0119277

**Published:** 2015-03-16

**Authors:** Johannes Steiner, Wen-Chih Wu, Matthew Jankowich, Bradley A. Maron, Satish Sharma, Gaurav Choudhary

**Affiliations:** 1 Vascular Research Laboratory, Providence VA Medical Center, Providence, Rhode Island, United States of America; 2 Department of Medicine, Warren Alpert Medical School of Brown University, Providence, Rhode Island, United States of America; 3 Veterans Affairs Boston Healthcare System, Department of Cardiology, Boston, Massachusetts, United States of America; 4 Brigham and Women’s Hospital and Harvard Medical School, Department of Internal Medicine, Division of Cardiovascular Medicine, Boston, Massachusetts, United States of America; VU University Medical Center, NETHERLANDS

## Abstract

**Objective:**

We aimed to identify the echocardiographic measures associated with survival in a patient population with a high prevalence of co-morbid cardiovascular and pulmonary disease that have significantly elevated estimated pulmonary artery systolic pressures (ePASP).

**Background:**

Pulmonary hypertension (PH) is a clinical feature of several cardiopulmonary diseases that are prevalent among elderly. While certain echocardiographic parameters have been shown to be important in the prognosis in specific PH groups, the prognostic relevance of echocardiographic characteristics in a cohort with multiple cardiopulmonary comorbidities is unclear.

**Methods:**

We retrospectively identified 152 patients with ePASP > 60 mmHg by echocardiography over a five year period (6/2006–11/2011) and followed until 4/2013. Candidate clinical and echocardiographic characteristics suggestive of PH severity were compared between deceased and surviving subpopulations. Cox proportional hazard modeling was used to identify echocardiographic predictors of death adjusted for age and clinical characteristics.

**Results:**

This was a predominantly elderly (age 78.8 ± 10.2 years), male (98.7%) cohort with several cardiopulmonary comorbidities. Overall mortality was high (69.7%, median survival 129 days). After adjusting for age and clinical characteristics, decreased right ventricular (RV) systolic function assessed by tricuspid annular plane systolic excursion (HR 0.56, 95% CI 0.33–0.96, p = 0.034) and increased RV thickness (HR: 4.34, 95% CI: 1.49–12.59, p = 0.007) were independently associated with mortality. In contrast, left ventricular systolic function, left ventricular diastolic parameters, ePASP, or echo-derived pulmonary vascular resistance (PVR) were not associated with increased mortality.

**Conclusion:**

In a cohort of patients with PH and high prevalence of cardio-pulmonary comorbidities, RV systolic function and hypertrophy are associated with mortality and may be the most relevant echocardiographic markers for prognosis.

## Introduction

Pulmonary hypertension (PH) is associated with several cardiopulmonary diseases that are prevalent among elderly patients [1–4]. Moreover, PH in patients with cardiopulmonary diseases is related to significant morbidity and mortality [5, 6].

There may be considerable overlap between the pathophysiologic mechanisms underlying PH in cardiopulmonary diseases. For example, in PH due to left heart disease, impaired left ventricular systolic and diastolic function and/or presence of significant mitral or aortic valvular disease can lead to left atrial hypertension and elevated pulmonary venous pressure. Persistently increased pulmonary venous pressures may lead to remodeling at the level of the pulmonary veins, capillaries, and arteries, ultimately resulting in elevated pulmonary vascular resistance (PVR). Similarly, while PH associated with lung diseases/hypoxia is associated with increased PVR, it is not uncommon to have concomitant left ventricular diastolic dysfunction and elevated left atrial pressure (LAP) in these diseases. An increase in pulmonary artery pressure (PAP) leads to an increased right ventricular (RV) afterload that results in RV hypertrophy. Eventually persistent PH causes RV dysfunction and RV failure [7–10]. Several of these pathophysiological mechanisms related to PH severity can be assessed using comprehensive echocardiography, a non-invasive and easily accessible modality [11].

However, reports of echocardiographic characteristics pertaining to left and right heart structure and function as well as the prognostic impact of these echocardiographically derived parameters related to mortality in a PH cohort with complex cardiopulmonary comorbidities are scant. Also, current survival prediction models for PH have not included any echocardiography derived parameters of biventricular geometry and function [12]. The objective of this study was to evaluate the prognostic relevance of echocardiographic indices in veteran patients with a high prevalence of cardiopulmonary diseases and PH, which could ultimately assist with treatment timing and potential targets. We hypothesized that PH effects on RV geometry and function are key determinates of survival in a patient population with multifactorial PH and sought to identify the echocardiographic indices that are associated with mortality in this patient population.

## Materials and Methods

### Study Population

The institutional review board at the Providence VA Medical Center approved the study. The Providence VAMC Institutional Review Board waived the requirement for informed consent for this minimal risk retrospective analysis. The study population was identified from the Providence VA echocardiography database that includes all echocardiograms performed at our institution. In this retrospective study, we identified 160 patients with reported estimated pulmonary artery systolic pressures (ePASP) > 60 mmHg on transthoracic echocardiography over a five year period (6/2006–11/2011). For patients with multiple studies, we included the first study performed and excluded subsequent studies. Eight patients had to be excluded from the final analysis due to missing standard 2D echocardiographic views.

### Clinical and Echocardiographic Data Collection

#### Clinical Characteristics

Clinical variables (demographics, past medical history, medications) and vital statistics data were retrospectively collected from electronic medical record. The clinical history was abstracted from the problem list in the patient’s electronic medical record and included history of systemic hypertension, chronic obstructive pulmonary disease, pulmonary embolus, pulmonary fibrosis, coronary artery disease, heart failure, and diabetes mellitus. The disposition of the patient at the time of echocardiogram was recorded as inpatient vs. outpatient.

#### Echocardiography

Routine echocardiographic assessment was performed on all patients, including M-mode, two-dimensional images, and color flow Doppler recording using Philips IE-33 with a 3.5-MHz transducer (Philips Medical Systems, Andover, MA). All the echocardiograms were reviewed again and all measurements were performed in accordance with the American Society of Echocardiography (ASE) /European Association of Echocardiography guidelines using the Phillips Xcelera Cardiac Reporting system. Heart rate was determined from the echocardiogram during the measurement of tricuspid regurgitant jet velocity. Estimated PA systolic pressure was determined by the sum of the transtricuspid gradient and estimated right atrial pressure based on inferior vena cava dimensions and respiratory variations. Diastolic function and LAP (elevated vs. normal) were assessed using the ASE recommended algorithm using a combination of echocardiographic variables including left atrial volume index (measured in apical four-chamber view by Simpson’s method of disc summation), transmitral E-wave acceleration, transmitral E-wave velocity (E), tissue Doppler at the septal and lateral mitral annulus (e’) [13]. Average E/e’ was calculated by using the average of septal and lateral e’ as the denominator. Systolic function was assessed by quantitative and/or visual evaluation of the left ventricular ejection fraction (LVEF). Significant left-sided valvular disease was defined as at least moderate stenosis or regurgitation in aortic and/or mitral valves. RV dimensions were measured in the apical 4-chamber view during diastole at the base and mid-cavity level. RV thickness was measured in the subcostal view in diastole. PVR was determined through the algorithm PVR = ratio of peak tricuspid regurgitant velocity to the right ventricular outflow tract time-velocity integral (TRV/TVI_RVOT_) x 10 + 0.16 in accordance with published guidelines [14]. RV function assessment included tricuspid annular plane systolic excursion (TAPSE), RV fractional area change (RV FAC), tricuspid annular velocities assessed by tissue Doppler imaging (RV S’).

Since M-Mode of tricuspid annulus was recorded only in a subset of patients (n = 56), we post-processed 2D 4-chamber images using a Java-based imaging software program (Image J, National Institute of Health) [15] to obtain M-Mode of the tricuspid annulus. These post-processed M-Mode images were then used to measure TAPSE (ppTAPSE) if the quality of M-mode was sufficient to measure TAPSE (as determined by two independent readers). Of the 152 post-processed images, 115 images had adequate quality to measure ppTAPSE. The ppTAPSE yielded a high correlation with the TAPSE measured from the M-mode images acquired at the time of echocardiogram (n = 56, correlation coefficient: 0.8251, p<0.001, mean difference 0.24 ± 0.31 cm). Representative example of TAPSE and ppTAPSE is displayed in the S1 Fig.

#### Outcome

The primary outcome measure was defined as all-cause mortality as assessed in April 2013 from the time of the index echocardiogram (first echocardiographic study with ePASP>60 mm Hg performed over the five year study period, time “0”).

### Analysis

Continuous variables were expressed as mean ± standard deviation, and compared using the unpaired Student t-test between the deceased and surviving cohorts. Categorical data were displayed as frequencies and percentages, and comparisons were made using Chi-square tests.

The relationship between echocardiographic indices reflecting LVEF, LV diastolic function (LAP, left atrial volume index, average E/e’), RV systolic function (ppTAPSE, RV S’, RV FAC), RV hypertrophy (RV thickness), ePASP and PVR; and mortality was investigated using Cox proportional hazards model. Mortality hazard ratios were adjusted for age alone or in a multivariate model containing the following covariates: age, gender, body mass index, systemic hypertension, chronic obstructive pulmonary disease, pulmonary embolus, pulmonary fibrosis, coronary artery disease, heart failure, and diabetes mellitus, inpatient status, heart rate, and significant left-sided valvular disease. For clinical applicability, we studied RV systolic function using ppTAPSE as a continuous variable as well as a categorical variable (≥1.8 vs <1.8 cm). The proportionality of the hazards function assumptions in the Cox model was examined by close observation of the Schoenfeld’s residuals plot over time and tested using the Schoenfeld Test in STATA. This test was not significant (p = 0.56) for ppTAPSE over time; thus, confirmed the proportionality of assumptions. In addition, age-adjusted Kaplan-Meier curves for ppTAPSE were generated and compared using the log-rank test.

Given that the main analysis used a novel TAPSE measurement technique (ppTAPSE), a sensitivity analysis was performed using the standard TAPSE recorded at the time of the study (n = 56) in lieu of ppTAPSE to estimate the adjusted hazard ratio of mortality.

All statistical tests were two sided. A p value <0.05 was considered statistically significant. All statistical analyses were performed using STATA 10 (StataCorp LP, College Station, TX).

## Results

### Study Population and Follow-up

**Fig 1:**
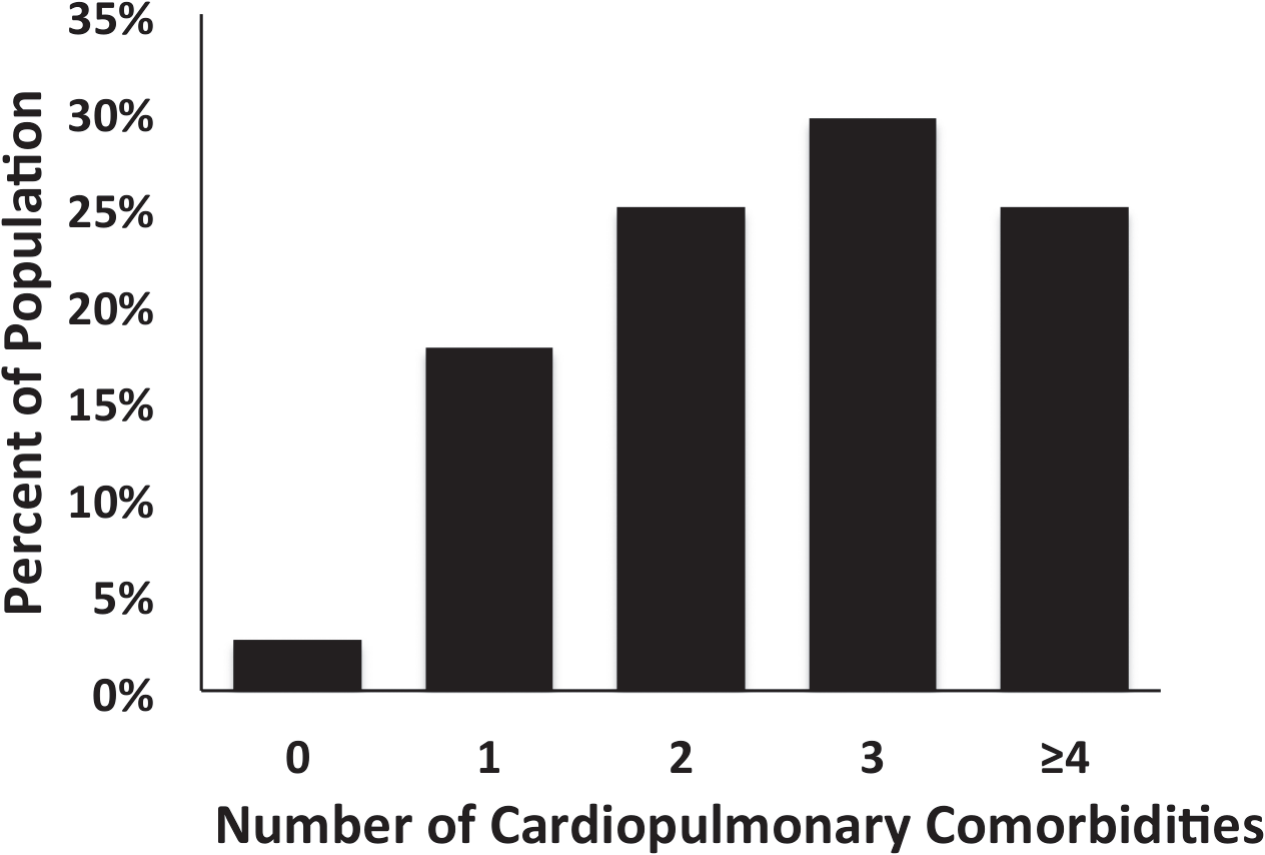
Distribution of cardiopulmonary comorbidities in patient cohort (n = 152), cardiopulmonary co-morbidities include greater than moderate left-sided valvular disease, coronary artery disease, heart failure, pulmonary embolus, chronic obstructive pulmonary disease and pulmonary fibrosis.

From a total of 4,565 individual patient echocardiograms screened, 160 patients (3.5%) met the predefined ePASP search criteria (i.e. ePASP ≥ 60 mm Hg), of which 152 patients had a complete echocardiographic dataset and were included in the analysis. The median follow-up time was 406 days. The clinical baseline characteristics of this cohort are summarized in Table 1. This was a predominantly elderly patient cohort (mean age 78.8 ± 10.2 years) with 98.7% males. Ninety seven percent of patients had at least one cardiopulmonary comorbidity as defined in Fig. 1. Prevalence of underlying coronary artery disease (52.6%), heart failure (51.3%), and valvular heart disease (46.7%) was high. Additionally, a significant proportion of patients carried pulmonary comorbidities including a history of chronic obstructive pulmonary disease (36.2%), pulmonary fibrosis (9.9%), and/or pulmonary embolus (2.6%). There was a significant overlap between cardiac and pulmonary comorbidities; e.g. 52.3% of patients diagnosed with chronic obstructive pulmonary disease also carried a history of coronary artery disease. About 57.6% of patients had their index echocardiogram performed in the inpatient setting.

**Table 1:** Baseline Clinical Characteristics.

Patient Characteristics	All patients (n = 152)	Alive (n = 46)	Deceased (n = 106)	p-value*
**Age (years)**	78.8 ± 10.2	80.1 ± 9.1	78.2 ± 10.6	0.28
**Males**	98.7%	100%	98.1%	NS
**Heart rate (beats/min)**	77.9 ± 15.7	72.1 ± 13.4	80.7 ± 16.6	<0.01
**Body mass index**	26.7 ± 5.7	27.7 ± 5.2	26.3 ± 5.8	0.16
**Inpatient**	57.6%	41.3%	64.8%	0.01
**Clinical history**				
**Heart failure**	51.3%	56.5%	49%	0.4
**Coronary artery Disease**	52.6%	50%	53.8%	0.67
**Hypertension**	75%	87%	69.8%	0.02
**Diabetes**	43.4%	37%	46.2%	0.29
**Valvular heart disease**	46.7%	50%	45.3%	0.6
**Atrial Fibrillation**	37.5%	30.4%	40.6%	0.24
**COPD**	36.2%	28.3%	39.6%	0.18
**Pulmonary Fibrosis**	9.9%	4.3%	12.3%	0.13
**Pulmonary Embolus**	2.6%	2.2%	2.8%	0.82
**Medications**				
**ACEIs**	39.5%	47.8%	35.8%	0.16
**ARBs**	11.8%	13%	11.3%	0.76
**Beta-blockers**	67.1%	80.4%	61.3%	0.02
**Warfarin**	28.1%	37%	25.5%	0.15
**Diuretics**	64.5%	67.4%	63.2%	0.62
**Nitrates**	20.4%	23.9%	18.9%	0.48

* Comparing alive vs. deceased.

There were 106 patients (69.7%) who had the primary outcome. The median survival was 129 days (Mean: 308 days, Range: 0–1,985 days). The patients who died at follow-up were more likely to have been inpatients at the time of the echocardiogram acquisition. The prevalence of cardiopulmonary comorbidities did not significantly differ between the groups, with the exception of hypertension, which is more prevalent in patients who were alive compared to those expired. Moreover, surviving patients were more likely to have lower heart rates and were more likely to be on beta-blockers (Table 1).

### Echocardiographic Characteristics

Table 2 shows echocardiographic parameters stratified by outcome. Analysis of echocardiography data in this cohort demonstrated an average ePASP of 68.1 ± 11.5 mmHg with evidence of substantial left heart and pulmonary vasculature remodeling: 87% displayed an increased left atrial volume index, 67.7% had elevated LAP, 41.5% had an LVEF < 55%, and 52.3% had an elevated PVR (>3 WU). The ePASP did not significantly relate to any of the other echocardiographic parameters (data not shown).

**Table 2:** Baseline Echocardiographic Characteristics.

Patient characteristics	All patients (n = 152)	Alive (n = 46)	Deceased (n = 106)	p-value*	N
**Echocardiographic dimensions**					
**LA Volume Index (mL/m^2^)**	41 ± 1.6	42.5 ± 2.8	40.4 ± 1.9	0.53	146
**RA Volume Index (mL/m^2^)**	38.2 ± 18.9	37.2 ± 16.5	38.6 ± 19.8	0.68	144
**RV Dimension base (cm)**	4.3 ± 0.9	4.3 ± 0.8	4.3 ± 1	0.71	141
**RV Dimension mid-cavity (cm)**	3.5 ± 0.9	3.4 ± 0.7	3.5 ± 1	0.62	141
**LV function**					
**LV Ejection Fraction (%)**	48.2 ± 15.1	48.1 ± 13.8	48.2 ± 15.7	0.98	152
**LV E Velocity (cm/s)**	107 ± 37	112 ± 35	102 ± 40	0.16	134
**LV A Velocity (cm/s)**	71 ± 37	75 ± 47	69 ± 31	0.48	100
**Septal e’ (cm/s)**	5.7 ± 2	5.5 ± 1.7	5.8 ± 2.1	0.44	100
**Lateral e’ (cm/s)**	8.4 ± 3.2	8.4 ± 2.9	8.4 ± 3.1	0.94	99
**Average E/e’**	17.36 ± 8.48	17.43 ± 6.53	17.32 ± 9.5	0.95	94
**E/A**	1.78 ± 1.01	1.98 ± 1.11	1.7 ± 0.96	0.2	100
**Elevated LAP (%)**	67.7	69.7	66.7	0.76	96
**RV function**					
**ppTAPSE (cm)**	1.6 ± 0.5	1.8 ± 0.6	1.5 ± 0.4	<0.01	115
**RV S’ (cm/s)**	6.5 ± 2.8	7.2 ± 3.3	5.8 ± 2	0.07	49
**RV e’ (cm/s)**	6.4 ± 2.7	6.1 ± 2.5	6.6 ± 2.9	0.49	48
**RV FAC (%)**	37 ± 14	39 ± 13	36 ± 14	0.25	140
**TR gradient (mmHg)**	60.1 ± 10.8	60.68 ± 10.6	59.9 ± 11	0.64	152
**RV thickness (cm)**	0.9 ± 0.2	0.8 ± 0.2	0.9 ± 0.2	0.03	130
**ePASP (mmHg)**	68.1 ± 11.5	70.2 ± 11	66.6 ± 11.1	0.07	152
**PVR (WU)**	3.2 ± 1.1	2.8 ± 1	3.3 ± 1.4	0.04	107

LAP = left atrial pressure; ppTAPSE = tricuspid annular plane systolic excursion measured on M-Mode images derived from Apical 4-chamber views using NIH ImageJ software; RV S′ = tissue Doppler systolic velocity at lateral tricuspid annulus; RV e’ = tissue Doppler diastolic velocity at lateral tricuspid annulus; FAC = fractional area change; ePASP = echo derived pulmonary artery systolic pressure; PVR = pulmonary vascular resistance.

* Comparing alive vs. deceased.

Patients with ePASP > = 60 mmHg who died were more likely to have reduced ppTAPSE (1.5 ± 0.4vs.1.8 ± 0.6, p < 0.01) and increased RV free wall thickness (0.9 ± 0.2 vs. 0.8 ± 0.2, p < 0.03). Also, the deceased patients were more likely to have an increased echo-derived PVR (3.3 ± 1.4vs.2.8 ± 1, p<0.04). On the other hand, LVEF, LAP, diastolic function indices, or RV FAC, did not differ between outcome groups.

### Survival Analysis

**Fig 2:**
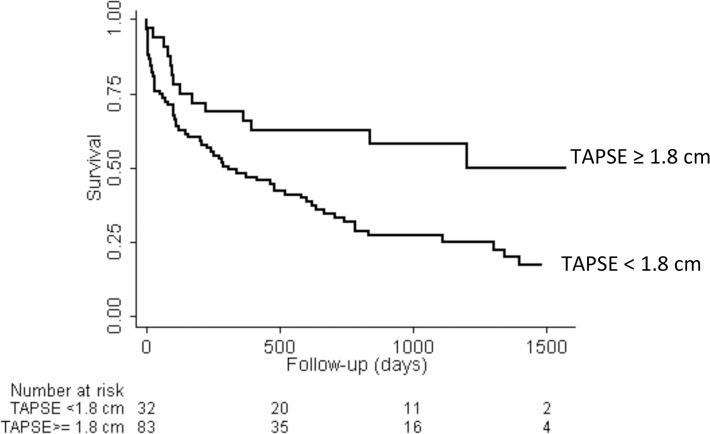
Kaplan Meier Survival Curves in patients with tricuspid annular plane systolic excursion ≥1.8 cam and < 1.8 cm (p = 0.006, log-rank test).

Table 3 shows the age- and multivariate-adjusted hazard ratios for death of echocardiographic indices. After adjusting for age and clinical confounders, ppTAPSE (Hazard Ratio 0.56/ cm increase in ppTAPSE, 95% CI: 0.33–0.96), and RV free wall thickness (Hazard Ratio: 4.34/ cm increase in RV free wall thickness, 95% CI: 1.49–12.59) were significantly associated with mortality risk. Fig. 2 shows the Kaplan-Meier survival curves for TAPSE <1.8 vs. > = 1.8 cm (p = 0.006). Sensitivity analysis using the TAPSE measured from M-Mode recorded at the time of the study (n = 56) showed consistent results with a hazard ratio of TAPSE in the multivariate model to be 0.40 (95% CI: 0.19–0.85).

**Table 3:** Hazard ratios.

Echo parameters	Age adjusted	95% Confidence Interval	Adjusted Model^#^	95% Confidence Interval
**LVEF (%)**	1.00	0.99–1.01		
**Avg E/e’**	1.00	0.97–1.03		
**LAP**	0.97	0.55–1.72		
**LA volume index (ml/m^2^)**	0.99	0.98–1.00		
**ppTAPSE (cm)**	0.60*	0.38–0.94	0.56*	0.33–0.96
**RV S’ (cm/s)**	0.90	0.79–1.04		
**RV FAC (%)**	0.60	0.13–2.76		
**RV thickness (cm)**	3.20*	1.22–8.35	4.34*	1.49–12.59
**ePASP (mm Hg)**	0.98	0.96–1.00		
**PVR> 3WU**	1.1	0.7–1.8		

* p<0.05

LVEF = Left ventricular ejection fraction; LAP = left atrial pressure; ppTAPSE = tricuspid annular plane systolic excursion measured on M-Mode images derived from Apical 4-chamber views using NIH ImageJ software; RV S' = tissue Doppler systolic velocity at lateral tricuspid annulus; FAC = fractional area change; RV e’ = tissue Doppler diastolic velocity at lateral tricuspid annulus; ePASP = echo derived pulmonary artery systolic pressure, PVR = pulmonary vascular resistance.

# Adjusted for age, gender, hypertension, COPD, pulmonary embolus, pulmonary fibrosis, body mass index, coronary artery disease, heart failure, diabetes, inpatient status, heart rate, greater than moderate left sided-valvular disease.

## Discussion

In the current study, we analyzed a cohort of patients with high ePASP who underwent routine echocardiography. In this elderly cohort with prevalent cardio-pulmonary comorbidities, we found that increased RV thickness and decreased RV systolic function as assessed by TAPSE represented the only routine echocardiography-derived marker associated with all-cause mortality.

The consequence of elevated pulmonary artery pressures is development of RV hypertrophy and right-sided HF. End-diastolic free wall RV thickness has been shown to be a marker of chronically high ePASP and PVR [16]. RV hypertrophy has been associated with worse outcomes in patients with PH associated with heart failure with preserved ejection fraction[17]. The significant relationship of RV thickness with survival in our cohort with multiple cardio-pulmonary comorbidities suggests that the prognostic role of RVH may extend beyond the population with heart failure alone. It is possible that increased RV thickness may be a better marker for long standing pulmonary hypertension than a single measurement of ePASP and identifies at-risk patients that develop adverse events.

The development of RV dysfunction is known to be associated with poor prognosis in patients with Pulmonary Arterial Hypertension [18], advanced heart failure [19], or chronic obstructive pulmonary disease[20, 21]. In advanced heart failure the association of PH with right ventricular dysfunction yielded a very poor prognosis whereas in the presence of PH and normal RV ejection fraction the prognosis was similar to that of the patients with normal pulmonary pressure [19]. Similarly, RV function and ePASP also predict prognosis independent of the severity of underlying chronic obstructive pulmonary disease [22]. Therefore, in PH, the degree of left-sided filling pressures, systolic dysfunction or presence of lung disease appear to be less relevant than the right ventricular response to the pressure overload. However, little is known about the effects of right ventricular geometry and function on overall mortality in multifactorial PH or PH in setting of multiple cardio-pulmonary comorbidities. Our results showed that RV function remained an important prognostic marker in this patient population. However, it remains to be seen if inclusion of these echocardiographic parameters would have any incremental value in the current survival prediction models for PH patients that included hemodynamic measurements [12].

Another important observation in our study was that not all indices of RV function perform the same in their association with mortality. While the ASE guidelines recommend using at least one index (FAC, TAPSE, myocardial performance index (MPI), or RV S) to assess RV systolic function on transthoracic echocardiogram [23], TAPSE has been shown to have the highest correlation with MRI derived RV ejection fraction[24]. This may explain our observation that TAPSE had significant association with mortality, while FAC, RV e’ and RV S’ did not. Thus, in this patient population, TAPSE assessment may be more meaningful as it is linearly associated with mortality risk. We chose a cut-off of 1.8 cm for categorical analysis based on prior literature[18]. However, other published thresholds for abnormal TAPSE include 1.4 cm [25] and 1.6 cm. [23] It remains to be seen if the categorical thresholds may differ in different disease cohorts and would need further validation in larger cohorts.

We found no significant association between ePASPs and mortality in our study cohort. In fact, a non-significant trend towards higher ePASP in the surviving cohort was observed. Lam et al described a significant relationship between increasing ePASP and mortality (unadjusted HR 4.65 per 10 mm Hg increase; p<0.001) in the general population [5]. Chronic heart failure [26] as well as hemodialysis [27] cohorts displayed a similar correlation with mortality and higher ePASPs. Our inclusion criteria, targeting only patients with severely increased ePASPs may offer one possible explanation for the lack of relationship between ePASP and mortality because PA pressures could decrease in the setting of RV failure [10]. Indeed, it has been reported that ePASP might represent a less important marker for PH severity and prognostication in a cohort with already advanced disease and decreasing cardiac output [28].

A significant proportion of our cohort displayed elevated PVR, suggesting pulmonary vascular remodeling that could be associated with the cardiopulmonary comorbidities. Although we observed a significant trend towards higher PVRs in the deceased cohort, echo-derived PVR was not independently associated with death after adjusting for clinical comorbidities. This might reflect limitations to the current echocardiographic estimation of PVR, and/or possible PVR underestimation in the presence of significant RV dysfunction[14, 29]. On the other hand, PVR seems to be in general less prognostically relevant than systolic RV function as suggested in previous right heart catheterization based literature [30].

### Limitations

First, the limitations of echocardiographic assessments of tricuspid regurgitation in PH patients are well described in the literature [31]. Although Doppler echocardiography may be inaccurate in estimating pulmonary artery pressure in patients being evaluated for PH [31], PASP > 50 mmHg on transthoracic echocardiography make the presence of significant underlying PH very likely according to current guidelines [32], Also, mean PAP can be accurately predicted from noninvasively estimated pulmonary artery systolic pressure over a wide pressure range for different etiologies of pulmonary hypertension [33]. Secondly, selection and referral bias, which are inherent to our retrospective study design, cannot be excluded despite our adjustment for potential confounders. However, the mortality in our cohort was similar to previously published community-based studies with multifactorial PH [2], and highlights the poor prognosis associated with the presence of PH in patients with cardiopulmonary comorbidities. Thirdly, classification into appropriate PH WHO groups was not possible due to the unavailability of right heart catheterization data in the majority of patients and the multitude of underlying comorbidities. The prevalence of underlying coronary artery disease, heart failure, valvular heart disease, associated cardiovascular risk factors, and chronic obstructive pulmonary disease was high in our cohort and suggests that cardiopulmonary diseases likely contributed to the majority of the elevated ePASPs as reported earlier [3]. However, none of the cardiopulmonary comorbidities differed significantly between outcome groups. In terms of clinical parameters, only in-patient status and higher heart rates were associated with poor survival, most likely as surrogates for higher acuity and lack of beta-blocker use; both of which were adjusted for in the analysis. The lack of invasive hemodynamics also limited our ability to compare the echocardiographic parameters with known indices of PH severity such as cardiac index and right atrial pressure which are important in the prognosis of patients with PH.

Also, since TAPSE recordings were available only in a subset of patients, we processed 2D 4-chamber images using a Java-based imaging software program (Image J, National Institute of Health) to obtain ppTAPSE, that showed very good correlation with original TAPSE; however, this method remains to be validated in other cohorts. Finally, although it is possible that the analysis of RV function using tissue cardiac MR or strain imaging could have provided further prognostic information, the possibility of stratifying the prognosis of PH patients using simple M-mode and tissue Doppler can be easily and widely applied in real world settings.

### Conclusion

Elevated ePASP in the elderly is associated with a high mortality. In a cohort of patients with high ePASP and high prevalence of co-morbid cardiovascular and pulmonary disease, RV systolic function and hypertrophy are associated with mortality and may be the prognostically most relevant echocardiographic markers.

## Supporting Information

S1 Fig Representative images showing M-Mode of the tricuspid annulus on the same patient derived using Image J (A) or acquired at time of study (B). (TIFF)
