# Supplementary figures and images for: Echocardiographic Predictors of Mortality in Patients with Pulmonary Hypertension and Cardiopulmonary Comorbidities
Source: PLoS One. 2015 Mar 16;10(3):e0119277. doi: 10.1371/journal.pone.0119277 (PMC4361665; doi:10.1371/journal.pone.0119277)

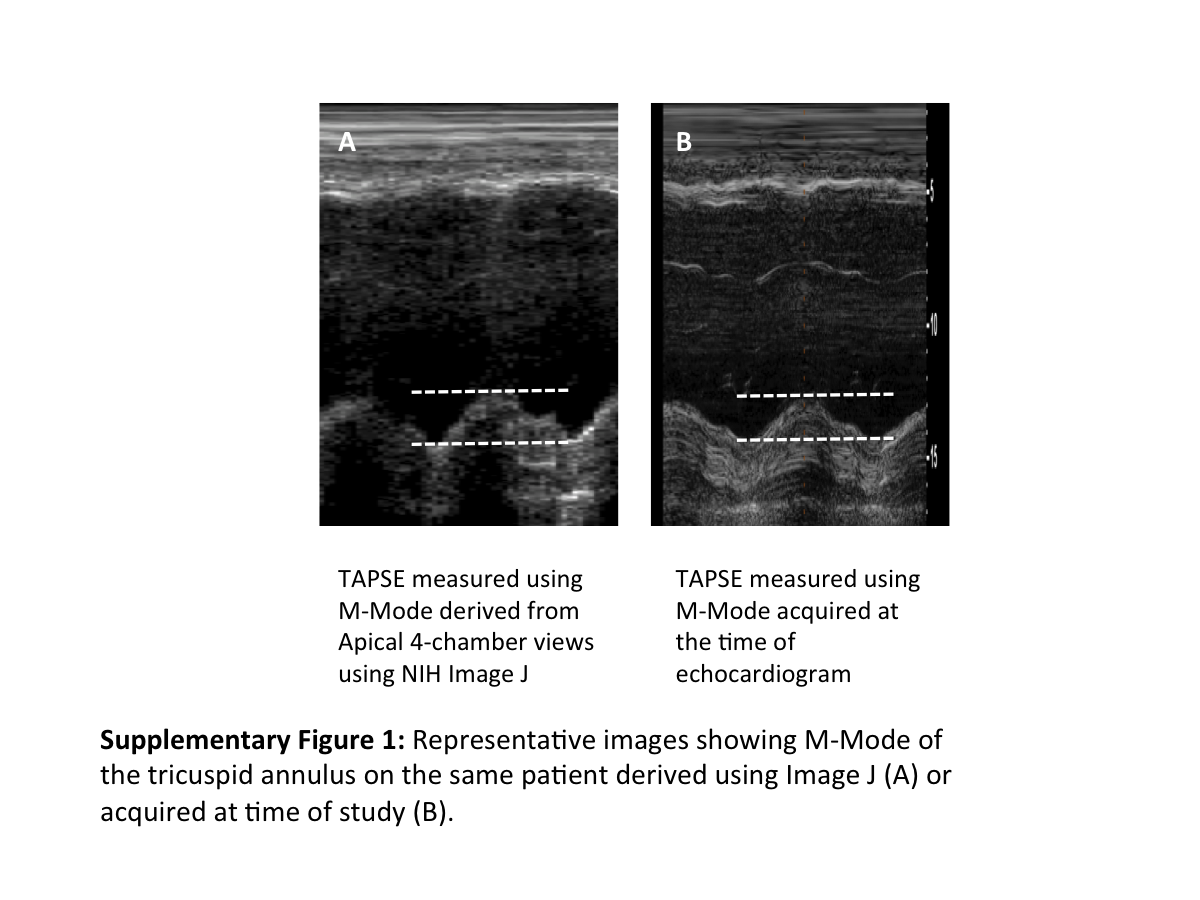

Supplement: S1 Fig — (TIFF) [file pone.0119277.s001.tiff]
